# Respiratory Viral Infection Prophylaxis and Treatment in the Transplant Population

**DOI:** 10.3390/v18010008

**Published:** 2025-12-20

**Authors:** Adriana A. M. Giuliani, Victor Chen, Nancy Law

**Affiliations:** 1 Division of Infectious Diseases and Global Public Health, Department of Medicine, University of California, La Jolla, CA 92093, USA; nalaw@health.ucsd.edu; 2 Division of Infectious Diseases, Rady Children’s Hospital, San Diego, CA 92123, USA; 3 Department of Pharmacy, University of California San Diego Health, La Jolla, CA 92093, USA; victorchen@health.ucsd.edu

**Keywords:** RSV, influenza, SARS-CoV-2, antiviral, vaccination, monoclonal antibody

## Abstract

Transplant patients experience high morbidity and mortality caused by respiratory viral infections (RVIs). In the past decade, numerous methods of prophylaxis and treatment have rapidly developed and continue to expand, with dozens of novel agents in preclinical and clinical trials. This includes recent scientific breakthroughs in virus structure, which have enabled the creation of respiratory syncytial virus (RSV) vaccines. While new vaccines, antivirals, monoclonal antibodies, and non-vaccine agents are becoming more available, their utility and safety in the transplant populations are often uncertain. This review summarizes the current landscape of RVIs in the transplant population, including approaches to pre- and post-exposure prophylaxis and treatment. We discuss the data behind vaccine timing, safety, and efficacy and current pre- and post-transplant recommendations, with a particular focus on influenza, SARS-CoV-2, and RSV. We also examine the potential benefits of antivirals, monoclonal antibodies, and novel agents used as prophylaxis, treatment, or adjuncts. While there remain many knowledge gaps, these new methods and ongoing advancements in RVI treatment and prevention promise to improve transplant patient outcomes.

## 1. Introduction and Epidemiology

**Figure 1:**
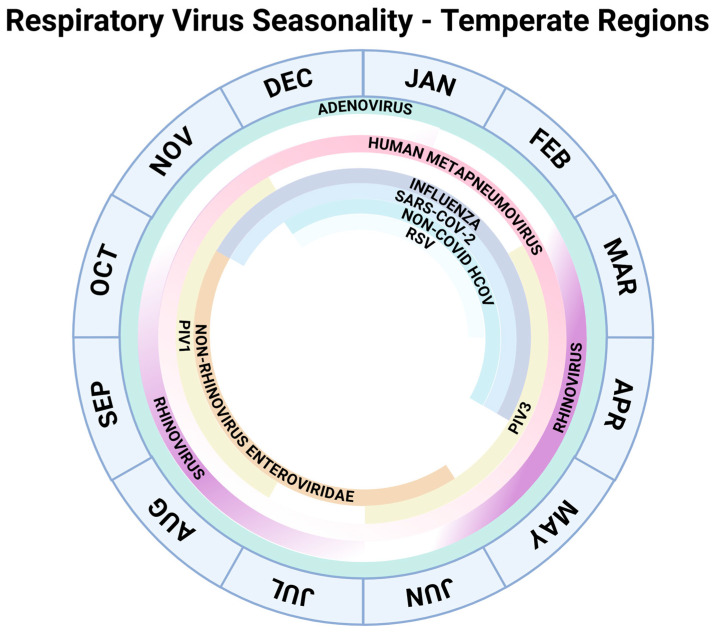
Seasonality of common respiratory viruses in temperate regions as included in this review [5,6,7].

Respiratory viral infections (RVIs) pose an unremitting risk to transplant recipients, an especially vulnerable population. Solid organ transplant (SOT) and hematopoietic stem cell transplant (HCT) recipients experience increased morbidity with RVIs, including an increased risk of acute and chronic graft dysfunction, severe lower respiratory tract disease, secondary bacterial infections, hospitalization, and mortality [1]. A retrospective study of allogeneic HCT recipients identified degree of immunosuppression as the most important factor in mortality rather than the properties of the different viruses themselves [2]. In addition to increased severity of disease, transplant recipients can exhibit prolonged viral shedding, which may lead to increased periods of contagiousness and risk of resistant variant emergence [3]. The timing of infection depends on virus seasonality, as depicted in Figure 1.

Decreasing vaccine rates and therefore decreased herd immunity to RVIs among the general population pose a significant risk to transplant patients [4]. Fortunately, the landscape of vaccines and novel RVI treatments is rapidly evolving. This review will discuss the current strategies for pre- and post-exposure prophylaxis and treatments available to transplant recipients, highlighting their efficacy, safety, and practical considerations in the immunocompromised population.

## 2. Influenza

### 2.1. Background

Despite vaccination being available since 1945 to stymie seasonal pandemics, the influenza virus remains a major cause of RVIs, related to its tendency to mutate via processes of antigenic drift and shift. A multicenter prospective study (2010–2015) of SOT and HCT recipients with confirmed influenza A found that patients most commonly presented with cough, fever, and coryza, although fever was absent in 36.2% of cases [8]. Seasonal vaccination reduced the risk of severe disease (pneumonia and intensive care unit [ICU] admission) and early antiviral therapy within 48 h improved outcomes [8]. SOT recipients (SOTRs) have a higher risk of complications than the general population, including pneumonia in up to 22–49% of cases, increased ICU admissions, increased mortality, and risks of allograft dysfunction and acute rejection [3]. These complications underline the importance of vaccination and early presentation for appropriate treatment.

### 2.2. Vaccination

#### 2.2.1. Available Vaccines

The inactivated influenza vaccine (IIV) is trivalent and directed towards two main types: influenza A (H1N1 and H3N2) and influenza B [9]. Annual vaccine strains are pre-selected in February in the Northern Hemisphere and in September in the Southern Hemisphere, based on those found in the global community.

Since its emergence in 1996, the highly pathogenic avian influenza (HPAI) A/H5N1 strain has remained a global public health concern. Ongoing research aims to develop effective countermeasures, including a universal influenza vaccine. Studies of split and adjuvanted whole-virus H5N1 vaccines have demonstrated safety and immunogenicity, while newer platforms such as virus-like particles (VLPs) and mRNA vaccines also show potential [10].

Although a live-attenuated influenza vaccine is available, it is contraindicated in immunosuppressed individuals. If administered, transplantation should be delayed for at least two weeks to minimize the risk of vaccine-derived viral replication [11].

#### 2.2.2. Timing

It is recommended to administer the IIV on an annual basis preceding the influenza season. Two doses separated by four weeks are required in previously unvaccinated patients less than nine-years-old. Pre-transplant administration is preferred.

For SOTRs in the post-transplant period, the vaccine can be given as early as one month after transplant [11]. HCT recipients are recommended to receive the vaccine at six months post-transplant although earlier vaccination at three months may be considered during periods of high transmission [12]. For patients with severe graft versus host disease (GVHD) or lymphopenia, a booster dose at four weeks may be considered [12].

#### 2.2.3. Efficacy and Safety

The immunogenicity of IIV in transplant recipients is consistently lower than in immunocompetent individuals- with seroprotection rates varying from 15–90% in SOTRs- and is variable depending on transplanted organ, level of immunosuppression, and vaccine composition [13]. Lung transplant recipients in particular were noted to have the lowest seropositive response rates [13].

The TRANSGRIPE 1–2 study demonstrated that a booster dose administered five weeks after the first dose in SOTRs was associated with higher seroconversion rate for H1N1 but not for other strains [14]. Recently, the STOP-FLU trial demonstrated improved vaccine response rate after administration of high dose (66%) or MF59-adjuvanted vaccine (60%) as compared to standard vaccine (42%) in SOTRs [15].

Despite reduced immunogenicity, the IIV is generally considered safe and has been associated with a decreased risk of complications including pneumonia, ICU admission, use of invasive ventilation, and death [3,8].

### 2.3. Pre- and Post-Exposure Prophylaxis

Pre-exposure prophylaxis with oseltamivir for 12 weeks may be an alternative for patients in whom the vaccine is not preferred or humoral response is expected to be diminished [3]. Post-exposure prophylaxis with oseltamivir for seven days is recommended in SOTRs who have had close contact with a patient with documented influenza [3].

### 2.4. Treatment

#### 2.4.1. Neuraminidase Inhibitors

Neuraminidase inhibitors (NAIs)- including oseltamivir, zanamivir, and peramivir- block the release of newly formed influenza virions from infected host cells [16]. Oseltamivir (Tamiflu) remains the preferred agent in immunocompromised patients due to its favorable safety profile and oral formulation [16]. Zanamivir (Relenza), administered by inhalation, is avoided in patients with chronic respiratory disease because of post-licensure reports of bronchospasm [16]. Peramivir (Rapivab), given intravenously, offers an alternative for patients unable to tolerate oral or inhaled medications and has demonstrated comparable efficacy, though data on its safety and optimal duration in immunocompromised populations remain limited [17]. In a multicenter study of SOT and HCT recipients with influenza infection, 94.1% received oseltamivir, and early treatment within 48 h was associated with improved outcomes [8]. Standard-dose oseltamivir was better tolerated than high-dose therapy, though higher doses were linked to lower viral resistance rates [18].

#### 2.4.2. Cap-Dependent Endonuclease Inhibitors

Cap-dependent endonuclease inhibitors (CDEIs) block a key step in viral mRNA synthesis. Baloxavir marboxil (Xofluza), approved for patients aged ≥5 years, is administered as a single dose and has shown efficacy comparable to oseltamivir with faster symptom resolution [18,19]. However, reduced viral susceptibility occurred in ~10% of cases, particularly with prolonged viral shedding, leading guidelines to discourage its routine use in immunocompromised patients [19]. Subsequent studies in high risk and immunocompromised individuals demonstrated similar efficacy to oseltamivir, and combination therapy with neuraminidase inhibitors was well tolerated but did not improve outcomes [8,20,21]. Novel CDEIs, including TG-1000, ZX-7101A, AL-794, and onradivir, show early promise but remain under investigation.

#### 2.4.3. CD388

CD388 is a long-acting injectable drug Fc conjugate containing multiple neuraminidase inhibitor copies, designed to protect against all known seasonal and pandemic influenza strains regardless of immune status [22]. In the Phase 2b NAVIGATE trial, a single injection demonstrated up to 76% efficacy in preventing laboratory-confirmed influenza over a 24-week season [22]. Protection was consistent across influenza A and B strains, and the drug was well tolerated with no significant safety concerns or dose-limiting adverse effects [22]. These findings highlight CD388’s potential as a durable, non-vaccine preventive strategy for individuals with impaired immunity, including transplant recipients.

Please refer to Table 1 for a summary of prophylactic and therapeutic options for the prevention and management of influenza in transplant recipients.

## 3. SARS-CoV-2

### 3.1. Background

SARS-CoV-2 infection carries significant morbidity and mortality in immunocompromised patients, with organ transplant recipients experiencing a 38% higher risk of death compared to the general population [24]. With advancements in early detection, treatment, and vaccination as well as changes in virility between variants, mortality in SOTRs has decreased from 20–25% at the beginning of the pandemic to 8–10% by 2022 [25]. However, transplant patients remain especially at risk of severe disease even with less virulent strains, given higher viral mutation rates and prolonged shedding [26]. Studies have shown increased ICU admission, mechanical ventilation, and mortality rates in SOTRs due to SARS-CoV-2 relative to other respiratory viruses [27]. Risk factors for severe disease include lung transplantation, recent rituximab or corticosteroid use, older age, and chronic comorbidities [28]. Vaccination with three or more doses and early antiviral treatment within seven days of symptom onset is protective, though vaccine effectiveness against hospitalization in immunocompromised adults ≥65 years was modest at 40% [29]. As with influenza, early vaccination and prompt antiviral therapy remain critical.

### 3.2. Vaccination

#### 3.2.1. Available Vaccines

Currently available vaccines include mRNA BNT162b2 (Cominarty, Pfizer-BioNTech—New York City, NY, USA), mRNA-1273 (Spikevax, Moderna—Cambridge, MA, USA), and the protein subunit vaccine NVX-CoV2373 (Novavax—Gaithersburg, MD, USA). The mRNA vaccines are preferred due to greater evidence of safety and efficacy in immunocompromised patients [30,31]. Normal dose vaccine is preferred; no improvement in immunogenicity was detected on using double dose in the RECOVAC trial [32].

#### 3.2.2. Timing

Immunocompromised individuals require a three-dose primary SARS-CoV-2 vaccine series due to poor humoral responses, with only 20–40% of transplant recipients developing antibodies after two doses compared to nearly 100% in healthy individuals [24,33]. Multiple studies in SOTRs show improved immunogenicity after three doses, though a subset remains nonresponsive [31,33]. Both homologous (same vaccine type) and heterologous (different vaccine type) third doses enhance antibody and T-cell responses, particularly in kidney transplant recipients [34]. The Centers for Disease Control and Prevention (CDC) recommends a fourth dose at least two months after the primary series; this further increases seropositivity (from 29.4% to 55.6%) and enhances neutralization activity against most variants, though Omicron responses remain suboptimal [35,36].

Vaccination should ideally be completed pre-transplant, but due to peri-transplant immunosuppression, the series should be repeated post-transplant, beginning at least one month after SOT [24,30]. For HCT recipients, vaccination is recommended at six months post-transplant, or as early as three months during community outbreaks [12]. Booster timing varies by region (6–12 months), with longer intervals improving hybrid immunity but increasing interim infection risk [37].

#### 3.2.3. Efficacy and Safety

Vaccine responses in immunocompromised patients are suboptimal but improve with additional doses, as shown in multiple meta-analyses [38]. Poor response is associated with older age, higher immunosuppression, and reduced renal function [33]. High-dose mycophenolate reduces humoral immunity in a dose-dependent manner [39]. While short-term mycophenolate interruption showed no benefit, studies suggest improved antibody responses when switching to everolimus or when mycophenolate is held for more than six weeks [32,40,41].

Despite low antibody titers, cross-protective cellular immunity may mitigate disease severity. Prior Omicron infection conferred T-cell–mediated protection against emerging variants such as JN.1 in SOTRs [42]. mRNA vaccines remain safe and well tolerated in both SOT and HCT recipients, with low rates of mild adverse effects [43].

### 3.3. Pre- and Post-Exposure Prophylaxis

Pemivibart (Pemgarda, Invivyd—New Haven, CT, USA) is a long-acting monoclonal IgG1 antibody authorized by the FDA for pre-exposure prophylaxis of SARS-CoV-2 in immunocompromised individuals aged ≥12 years [44,45]. While not a substitute for vaccination or treatment of active infection, it may be administered every three months; however, clinical benefit in this population remains uncertain [44].

### 3.4. Treatment

#### 3.4.1. Remdesivir (Veklury, Gilead Sciences—Foster City, CA, USA)

Remdesivir is a nucleotide analogue inhibiting the RNA-dependent RNA polymerase in SARS-CoV-2. A large retrospective review of immunocompromised adults demonstrated improved mortality at 14 and 28 days, with an approximately 35% lower mortality risk in SOT and HCT recipients [46]. Early initiation within seven days of symptom onset in outpatient SOTRs is associated with decreased hospitalization rate, with an adjusted hazard ratio of 0.12 [47]. It is the preferred method of treatment in hospitalized immunocompromised individuals.

#### 3.4.2. Nirmatrelvir-Ritonavir (Paxlovid, Pfizer—New York City, NY, USA)

Nirmatrelvir inhibits the SARS-CoV-2 main protease while ritonavir boosts the drug plasma levels via CYP3A inhibition [48]. Approved for early treatment of mild-to-moderate COVID-19 in high-risk patients, extended 10–15 day courses in immunocompromised hosts modestly reduced viral rebound without shortening viral shedding [49]. Use in transplant patients is limited by major drug-drug interactions with calcineurin and mTOR inhibitors [48].

#### 3.4.3. Molnupiravir (Lagevrio, Merck & Co., Ltd.—Rahway, NJ, USA)

Molnupiravir, an oral nucleoside analogue, is authorized for mild-to-moderate SARS-CoV-2 infection in adults at risk for severe disease [50]. It is generally well tolerated and has been associated with reduced hospitalization rates among SOTRs, though efficacy appears lower than with remdesivir or nirmatrelvir-ritonavir [50]. Molnupiravir is considered teratogenic and the package insert recommends reliable contraception at least four days in females and for three months in males after completion of therapy [51].

#### 3.4.4. Monoclonal Antibodies

Anti-SARS-CoV-2 monoclonal antibodies (mAbs) have been used effectively in SOTRs for pre-exposure and post-exposure prophylaxis and early treatment of mild-to-moderate infection. No evidence of rejection has been observed in observational studies. However, many mAbs have become less efficacious with the development of new variants [33].

Please refer to Table 2 for an overview of SARS-CoV-2 vaccines, monoclonal antibodies, and antiviral agents utilized for prevention and treatment in transplant recipients.

## 4. Respiratory Syncytial Virus (RSV)

### 4.1. Background

RSV causes substantial morbidity in transplant recipients, with high rates of lower respiratory tract disease, respiratory failure, graft dysfunction, and mortality. In lung transplant recipients, RSV infection is linked to a 29% incidence of new allograft dysfunction within three months [51]. Early vaccine development was hindered by enhanced respiratory disease (ERD) observed with a formalin-inactivated vaccine in the 1960s which increased the severity of illness caused by natural infection [52]. Recent advances in knowledge of the RSV pre-fusion (pre-F) protein structure and streamlined vaccine pathways following the SARS-CoV-2 pandemic have revitalized RSV prevention efforts, resulting in three approved adult vaccines and over thirty candidates in development [53].

### 4.2. Vaccination

#### 4.2.1. Available Vaccines

Current RSV vaccines target the pre-F protein, including two protein subunit vaccines, Abrysvo (non-adjuvanted, Pfizer—New York City, NY, USA) and Arexvy (adjuvanted, GSK—London, UK), and one mRNA vaccine, mRESVIA (Moderna—Cambridge, MA, USA) [54]. Abrysvo is approved for adults ≥18 years and for use during pregnancy to protect infants [54]. Arexvy is indicated for at-risk adults ≥50 years or ≥60 years regardless of risk [54]. mRESVIA may be used in adults ≥18 years at high risk, including immunocompromised and transplant recipients, and in all adults ≥60 years [55].

#### 4.2.2. Timing

A single dose of RSV vaccine is recommended prior to the RSV season, ideally before transplantation. Post-transplant vaccination lacks definitive guidance, though experts suggest delaying for three to six months [12]. Revaccination is not routinely advised but may be considered in non-responders [56]. Early data indicate that booster doses offer limited additional benefit in transplant recipients [56,57].

#### 4.2.3. Efficacy and Safety

Ongoing studies continue to evaluate RSV vaccine efficacy and safety in transplant recipients. Vaccine effectiveness appears reduced compared to the general population (50–69% vs. 75%), with the lowest rates among stem cell transplant recipients (29–44%) [58]. Antibody persistence up to one year post-vaccination has been observed, particularly in those further from transplant, off immunosuppression, and with higher lymphocyte counts [59]. Despite modest seroconversion rates, robust CD4+ T-cell responses suggest potential clinical benefit [60,61]. The adjuvanted Arexvy vaccine may enhance immunogenicity compared to the non-adjuvanted Abrysvo vaccine [36]. There is limited data for the use of mRESVIA in the immunocompromised population and it is not currently recommended [56]. Mycophenolate use reduces vaccine response, though optimal management is undefined [61]. Safety data show no transplant-related rejection or serious immune complications, with a small risk of Guillain-Barré syndrome (~11.2 per million doses) with Abrysvo and Arexvy [58,61].

### 4.3. Pre-Exposure Prophylaxis

Palivizumab (Synagis, Sobi—Waltham, MA, USA), an RSV fusion protein-targeted mAb requiring monthly administration, was previously used off-label in high-risk adults but showed no clear clinical benefit in transplant populations [62,63]. It has been replaced by newer long-acting antibodies and is expected to be discontinued after 2025 [64].

Next-generation RSV mAbs incorporate Fc modifications that prolong half-life, enabling single-dose seasonal protection [53]. Nirsevimab (Beyfortus, SANOFI—Swiftwater, PA, USA) provides ~70% efficacy against RSV-related lower respiratory tract infection in infants, though adult dosing remains undefined [53]. Clesrovimab (Enflonsia, Merck & Co., Ltd.—Rahway, NJ, USA), a similar long-acting candidate, shows comparable duration and efficacy [53]. Both agents hold promise for immunocompromised and transplant patients, but clinical data in these groups are currently lacking. Although mAb-resistant RSV variants have been reported, they generally exhibit reduced viral fitness [53].

These emerging long-acting monoclonal antibodies represent an important step toward passive immunoprophylaxis for vulnerable populations and complement ongoing efforts to optimize vaccine-based prevention strategies in transplant recipients.

### 4.4. Treatment

#### 4.4.1. Ribavirin

Ribavirin (multiple brand names and manufacturers), a nucleoside analog approved for treatment of RSV lower respiratory tract disease in high-risk and transplant populations, has shown variable benefit [3]. Early data in HCT recipients suggested reduced disease progression and mortality when combined with immunomodulators such as palivizumab, IVIG, or RSV-IVIG [3]. However, more recent systematic reviews in lung transplant recipients report conflicting efficacy, with no significant association between ribavirin use and reduced chronic lung allograft dysfunction [65].

Aerosolized ribavirin remains the preferred route due to higher bronchoalveolar concentrations compared with oral formulations [66]. While oral ribavirin may serve as a feasible alternative in resource-limited settings or when inhaled therapy is impractical, evidence supporting its efficacy remains limited [67]. It should be noted that ribavirin is highly teratogenic and is classified as Category X by the FDA [68].

#### 4.4.2. Molnupiravir (Lagevrio, Merck & Co., Ltd.—Rahway, NJ, USA)

Similar to its use in SARS-CoV-2, Molnupiravir may have some activity against RSV however clinical data in immunocompromised patients is lacking. A recent study of healthy adults did not meet primary efficacy endpoints of quantitative viral culture however did show modest non-significant benefits with treatment including rapid improvement in symptoms [69].

Please refer to Table 3 for a summary of available RSV vaccines and monoclonal antibody products for prevention and management in transplant recipients.

## 5. Other Respiratory Viruses

Vaccines for rhinovirus/enterovirus, parainfluenza virus (PIV), human metapneumovirus (hMPV), adenovirus (HAdV), and non-COVID human coronaviruses (HCoV) are in early development. Cytomegalovirus (CMV), while not strictly a respiratory virus, is known to cause severe respiratory disease in transplant patients. High-risk and transplant populations remain underrepresented in trials, and progress is hindered by antigenic diversity, rapid mutation, limited correlates of protection, and weak commercial incentives for low-morbidity pathogens.

### 5.1. Cytomegalovirus (CMV)

Cytomegalovirus (CMV) is a common post-transplant opportunistic infection; CMV pneumonia remains a rare but severe complication with approximately 60% one-year mortality in HCT recipients despite advances in antiviral therapy [71]. Highest risk groups include seropositive HCT recipients and seronegative SOTRs receiving organs from seropositive donors [72]. Prevention relies on antiviral prophylaxis or preemptive therapy guided by CMV PCR surveillance [72]. Valganciclovir (Valcyte) and letermovir (Prevymix) are principal agents used for CMV prophylaxis, with valganciclovir standard for most SOTRs (3–6-month duration, up to 12 months in lung transplantation) and letermovir approved for allogeneic HCT and kidney transplant recipients, having demonstrated non-inferiority and fewer adverse effects [73]. In HCT recipients, preemptive therapy based on center-specific PCR thresholds is commonly employed [72]. CMV pneumonia is typically treated with intravenous ganciclovir, with adjunctive immunoglobulin sometimes used despite limited supporting evidence [74]. Alternative or combination therapies, including foscarnet (Foscavir) and cidofovir (Vistide) can be considered [74]. Although maribavir (Livtencity) is effective for refractory or resistant CMV infection, it is not preferred for invasive disease including pneumonia [75]. CMV-specific adoptive T-cell therapy is an emerging strategy for prophylaxis and treatment of refractory disease [76]. Vaccines remain under development, although none are currently approved [76].

### 5.2. Rhinovirus/Enterovirus

Vaccine development is limited by >150 rhinovirus serotypes [77]. Experimental mRNA platforms show cross-reactive immunity in animals [78]. Several antivirals including pleconaril, rupintrivir, and remdesivir, demonstrate in vitro activity but lack clinical evidence [79].

### 5.3. Human Metapneumovirus (hMPV)

No approved therapies exist. Early trials of combined RSV/hMPV/PIV-3 (Sanofi) and mRNA vaccines (Moderna) demonstrate safety and immunogenicity [77,80]. Investigational mAbs and T-cell-based therapies show promise in preclinical studies [81]. There is limited evidence for the efficacy of probenecid but further studies are needed [81].

### 5.4. Adenovirus (HAdV)

The AdV-4/7 vaccine, covering serotypes 4 and 7, two of the most prominent causing respiratory disease, provides durable protection but is restricted to military use [82]. Cidofovir remains standard therapy, with brincidofovir under investigation as a safer alternative [83].

### 5.5. Non-COVID Human Coronavirus (HCoV)

Multiple preclinical and early clinical vaccines are under investigation, though durable immunity remains a challenge [84]. No antiviral therapies are approved [84].

### 5.6. Non-RSV Paramyxoviridae (e.g., Parainfluenza [PIV])

Novel antivirals under study include GHP-88309 and DAS-181, the latter showing post hoc benefit in immunocompromised patients [85,86]. Ribavirin is sometimes used off-label for severe cases, though efficacy data are limited [87].

## 6. Future Directions

Transplant recipients remain underrepresented in antiviral and vaccine trials, limiting evidence and delaying guidance. While novel agents, some described in Table 4, are promising, few are tested in transplant or immunocompromised populations. Dedicated transplant-specific studies are needed to define optimal dosing, timing, and safety. Long-acting monoclonal antibodies and non-vaccine-based prophylaxis may overcome poor vaccine immunogenicity in this population. Continued viral surveillance and public vaccine advocacy remain essential amid evolving variants and declining vaccination rates.

## 7. Conclusions

Pre- and post-exposure prophylaxis are central to mitigating respiratory viral infection risk in transplant recipients. Pre- and post-exposure prophylaxis strategies are summarized in Table 5. Emerging long-acting and variant-targeted agents show promise but require further validation in transplant-specific populations. Vaccination remains foundational, complemented by antivirals and monoclonal antibodies.

## Figures and Tables

**Table 1:** Influenza Vaccines and Antivirals in Transplant Recipients.

Agent	Class/Mechanism	Dosing & Schedule	Timing	Clinical/Efficacy	Safety
Fluzone High-Dose	HD-IIV3 (inactivated)	60 µg hemagglutinin/0.5 mL	Annual; pre-Tx ^1^ preferred [15]; ≥1 month post-SOT [11], ≥3–6 month post-HCT [12]	↑ Seroconversion vs. standard; ↓ pneumonia, ICU, mortality [8]	Safe; mild AEs ^2^
Fluad	aIIV3 (adjuvanted)	15 µg hemagglutinin/0.5 mL	Same as above	MF59 adjuvant enhances immune response [15]	Well-tolerated
Oseltamivir (Tamiflu)	Neuraminidase inhibitor	75 mg PO daily ×12 weeks (pre-exp) or ×7 days (post-exp) [3]	Pre-exposure if vaccine suboptimal [3]; post-exposure as treatment	↓ Influenza complications; improved outcomes if given within 48 h [8]	Safe; renally adjust
Baloxavir marboxil (Xofluza)	Cap-dependent endonuclease inhibitor	Single PO dose, consider in combo with NAI [19]	Early outpatient (<48 h) [19]	Comparable efficacy to oseltamivir; ↑ resistance risk [22]	Limited data in SOT
CD388	Investigational neuraminidase inhibitor conjugate	Single injection (24 week efficacy) [23]	Seasonal pre-exposure	76% efficacy in healthy adults; non-vaccine option [23]	Phase 2b; well tolerated [23]

^1^ “Tx”—transplant. ^2^ “AEs”—adverse effects.

**Table 2:** SARS-CoV-2 Vaccines, Monoclonal Antibodies, and Antivirals.

Agent	Class/Mechanism	Dosing & Schedule	Timing	Clinical/Efficacy	Safety
mRNA-1273 (Spikevax)	mRNA vaccine	4-dose (0, 4 weeks, +4 weeks, 6 month booster)	Pre-Tx ^1^ preferred but should also repeat post-Tx ^1^; ≥1 month post-SOT [28], ≥3 months post-HCT [24]	20–40% seroconversion after 2 doses; improved ≥3 [31,33]	No rejection/GVHD ^2^ [31,43]
mRNA BNT162b2 (Comirnaty)	mRNA vaccine	4-dose (0, 3 weeks, +4 weeks, 6 month booster)	Same as above	Boosters improve humoral response [38]	Safe in SOT/HCT [24]
Pemivibart (Pemgarda)	Long-acting mAb	4500 mg IV q 3 months	Pre-Tx ^1^ preferred	↓ Hospitalization; variant-dependent [44]	EUA ^3^, limited data
Remdesivir (Veklury)	RNA-polymerase inhibitor	3–5 days IV	Early (<7 days) outpatient or inpatient [47]	↓ Hospitalization/mortality [46,47]	Safe; monitor renal function
Nirmatrelvir-Ritonavir (Paxlovid)	Protease inhibitor combination	5–15 days PO	Early (<5 days) [49]	↓ Viral load; monitor DDIs ^4^	CYP3A interaction risk [48]
Molnupiravir (Lagevrio)	Nucleoside analog	5 days PO	Early (<5 days) [50]	↓ Hospitalization; less effective than remdesivir and nirmatrelvir-ritonavir [50]	Well-tolerated

^1^ “Tx”—transplant. ^2^ “GVHD”—graft versus host disease. ^3^ “EUA”—emergency use authorization. ^4^ “DDIs”—drug drug interactions.

**Table 3:** RSV Vaccines and Monoclonal Antibodies.

Agent	Class/Mechanism	Dosing	Timing	Clinical/Efficacy	Safety
Abrysvo	Protein subunit (non-adjuvanted) vaccine	Single pre-season dose [54]	Pre-Tx ^1^ preferred; ≥3–6 months post-Tx ^1^ [12,61]	50–69% VE ^2^; 1-year antibody persistence; Lower response in HCT (29–44%) [58,59]	Rare GBS ^3^; no rejection [58,61]
Arexvy	Protein subunit (adjuvanted) vaccine	Same as above	Same as above	50–69% VE ^2^; improved CD4+ response; preferred for immunocompromised [61]	Rare GBS ^3^; mild AEs ^4^ [58]
mRESVIA	mRNA vaccine	Same as above, not preferred	Same as above, not preferred	Potential in high-risk SOT [56]	Safe in PLWH ^5^; awaiting SOT data [70]
Nirsevimab	Long-acting mAb (F-protein)	Single IM pre-season, adult dose unknown	No adult data	70% efficacy in infants; no adult data; potential SOT use [53]	Safe in infants

^1^ “Tx”—transplant. ^2^ “VE”—vaccine efficacy. ^3^ “GBS”—Guillain-Barré Syndrome. ^4^ “AEs”—adverse effects. ^5^ “PLWH”—persons living with HIV.

**Table 4:** Novel Agents Under Investigation.

Virus	Agent	Mechanism	Stage	Findings	Status in Transplant
SARS-CoV-2	Ibuzatrelvir	Protease inhibitor	Phase 2	↓ Viral load [88]	No data
SARS-CoV-2	Ensitrelvir	Protease inhibitor	Phase 3	↓ Symptoms [89]	Potential benefit in IC ^1^ [89]
SARS-CoV-2	Obeldesivir	RNA pol inhibitor	Phase 3	↓ Symptoms [90]	No data
SARS-CoV-2	Bemnifosbuvir	RNA pol inhibitor	Phase 2	↓ Viral load [91]	No data
SARS-CoV-2	BIT225	Envelope protein inhibitor	Phase 2	Did not meet primary efficacy endpoint [92]	Investigational
RSV	Ribavirin	Nucleoside analog (inhaled preferred)	Approved	↓ LRTI progression ± IVIG/mAb, ?effect in lung Tx ^2^ [3,66]	Standard care
RSV	Molnupiravir	Nucleoside analog	Approved	↓ Symptoms [69]	No data
RSV	EDP-938	Nucleoprotein inhibitor	Phase 2	↓ Viral load/symptoms [93,94]	No data
RSV	ALN-RSV01	siRNA (inhaled)	Phase 2	↓ BOS ^3^ in lung Tx ^2^; safe [95]	Promising adjunct
RSV	RI-001 and RI-002	RSV Ab-rich IVIG	Approved	Early use may improve outcomes in IC [96]	Potential benefit in HCT, no SOT data [96]
Other RVI	DAS-181	Sialidase fusion protein (inhaled)	Phase 2	Benefit in select immunocompromised [86]	Investigational
Other RVI	T-cell adoptive therapy	Seropositive donor T cells	Phase 1–2	↓ Viral load, improve survival [97]	Investigational, promising in HCT

^1^ “IC”—immunocompromised. ^2^ “Tx”—transplant. ^3^ “BOS”—bronchiolitis obliterans syndrome.

**Table 5:** Comparative Pre-Exposure and Post-Exposure Prophylaxis Strategies.

Virus	Pre-Exposure	PrEP ^1^ Timing	Post-Exposure	PEP ^2^ Timing	Notes
Influenza	Annual IIV; oseltamivir ×12 weeks	Annual vaccine: ≥1 months post-SOT, ≥3–6 months post-HCT	Oseltamivir ×7 days	Best within 48 h	Vaccine cornerstone; antiviral bridge
SARS-CoV-2	mRNA booster vaccines; Pemivibart q 3 months	Boosters plus annual vaccine, timing same as above	Remdesivir, nirmatrelvir-ritonavir, molnupiravir	Early within 7 days	Variant-dependent prophylaxis
RSV	Single vaccine; mAb	Annual pre-season	Ribavirin ± IVIG/mAb	Early	Adjuvanted vaccines enhance immunity
Other RVI	Trial vaccines (hMPV, PIV)	Vaccine-specific	Cidofovir, ribavirin	Case-specific	Encourage trial enrollment

^1^ “PrEP”—pre-exposure prophylaxis. ^2^ “PEP”—post-exposure prophylaxis.

## Data Availability

No new data were created or analyzed in this study.

## Abbreviations

The following abbreviations are used in this manuscript:

RVI	Respiratory viral infection
SOT	Solid organ transplant
HCT	Hematopoietic stem cell transplant
SOTR	Solid organ transplant recipient
RSV	Respiratory syncytial virus
mAb	Monoclonal antibody
